# Discrimination and sexual harassment – Results from the largest German medical university

**DOI:** 10.1093/eurpub/ckac131.276

**Published:** 2022-10-25

**Authors:** S Ludwig, S Jenner, R Berger, S Tappert, C Kurmeyer, S Oertelt-Prigione, M Petzold

**Affiliations:** 1 Charité - Universitätsmedizin Berlin, Berlin, Germany; 2 Catholic University of Applied Sciences, Mainz, Germany; 3 Bielefeld University, Bielefeld, Germany; 4 Radboud University, Nijmegen, Netherlands

## Abstract

**Background:**

Discrimination and sexual harassment in the workplace and in higher education institutions are important public health issues. Here we aim at analyzing the prevalence of discrimination and sexual harassment of lecturers and students at one of the largest teaching hospitals in Europe. We assess whether there are differences between lecturers and students, women and men, and different study programs.

**Methods:**

An online questionnaire was sent to N = 7095 students of all study programs and N = 2528 lecturers at Charité - Universitätsmedizin Berlin. The survey was conducted from November 2018 to February 2019. We investigated experienced or observed discrimination or sexual harassment at the medical faculty. Furthermore, we analyzed frequency, perpetrators, situational factors, attributed reasons and forms of harassment encountered.

**Results:**

A total of 964 (14%) students (S) and 275 (11%) of lecturers (L) participated in the survey. Discriminatory behavior was witnessed and/or experienced by 49,6% of students (L: 31%), sexual harassment by 23,6% of students (L: 19,2%). Students state lecturers (85,9%) as main source of discriminatory behavior (L: directors/supervisors: 47,4%; students 41,0%). Sex/Gender (S: 71%; L: 60,3%) is cited most frequently as reason for discriminatory experiences. Female students and faculty experience more discrimination and sexual harassment.

**Conclusions:**

Discrimination and sexual harassment are prevalent in academic medicine. There are differences in the reasons and sources of discrimination and sexual harassment between students and lecturers. Specific programs for lecturers and students are necessary to educate the faculty on how to prevent and respond to it and whom to address.

**Key messages:**

• National preventive strategies should be implemented to tackle issues of discrimination and harassment in higher education institutions.

• Special attention should be paid to female students and lecturers.

